# Genetic variations (eQTLs) in muscle transcriptome and mitochondrial genes, and trans-eQTL molecular pathways in feed efficiency from Danish breeding pigs

**DOI:** 10.1371/journal.pone.0239143

**Published:** 2020-09-17

**Authors:** Victor A. O. Carmelo, Haja N. Kadarmideen

**Affiliations:** Quantitative Genomics, Bioinformatics and Computational Biology Group, Department of Applied Mathematics and Computer Science, Technical University of Denmark, Kongens Lyngby, Denmark; University of Illinois, UNITED STATES

## Abstract

Feed efficiency (FE) is a key trait in pig production, as improvement in FE has positive economic and environmental impact. FE is a complex phenotype and testing animals for FE is costly. Therefore, there has been a desire to find functionally relevant single nucleotide polymorphisms (SNPs) as biomarkers, to improve our biological understanding of FE as well as accuracy of genomic prediction for FE. We have performed a cis- and trans- eQTL (expression quantitative trait loci) analysis, in a population of Danbred Durocs (N = 11) and Danbred Landrace (N = 27) using both a linear and ANOVA model based on muscle tissue RNA-seq. We analyzed a total of 1425x19179 or 2.7x10^7^ Gene-SNP combinations in eQTL detection models for FE. The 1425 genes were from RNA-Seq based differential gene expression analyses using 25880 genes related to FE and additionally combined with mitochondrial genes. The 19179 SNPs were from applying stringent quality control and linkage disequilibrium filtering on genotype data using a GGP Porcine HD 70k SNP array. We applied 1000 fold bootstrapping and enrichment analysis to further validate and analyze our detected eQTLs. We identified 13 eQTLs with FDR < 0.1, affecting several genes found in previous studies of commercial pig breeds. Examples include MYO19, CPT1B, ACSL1, IER5L, CPT1A, SUCLA2, CSRNP1, PARK7 and MFF. The bootstrapping results showed statistically significant enrichment (p-value<2.2x10^-16^) of eQTLs with p-value < 0.01 in both cis and trans-eQTLs. Enrichment analysis of top trans-eQTLs revealed high enrichment for gene categories and gene ontologies associated with genomic context and expression regulation. This included transcription factors (p-value = 1.0x10^-13^), DNA-binding (GO:0003677, p-value = 8.9x10^-14^), DNA-binding transcription factor activity (GO:0003700,) nucleus gene (GO:0005634, p-value<2.2x10^-16^), negative regulation of expression (GO:0010629, p-value<2.2x10^-16^). These results would be useful for future genome assisted breeding of pigs to improve FE, and in the improved understanding of the functional mechanism of trans eQTLs.

## Introduction

The biological background of complex traits is expressed through molecular processes triggered by a combination of genetics, epigenetics and the environment. [1]. Almost per definition, complex traits are controlled by multiple genetic variants [2–4], which further complicates the causal structure between genetic variants and complex traits. While ample genetic markers have been identified for complex traits, the understanding of the functional effect of identified genetic markers is challenging to identify. One way of tackling this issue, is identify correlations between genetics and gene expression, thus identifying a direct effect of genetic variation. This allows for a straightforward interpretation of the effect of genetic variation based on pathway and functional knowledge of implicated genes. This can be done through the identification of eQTLs, mapping genetic variants that influence gene expression patterns of genes in various tissues, originally termed as systems genetics [5, 6]. The usage of both genetic and transcriptomic information, combined with pathway and phenotype data can be a powerful way of identifying biomarkers for traits of interest.

There are number of eQTL studies in pigs using high density SNP array data and RNA-Seq based transcriptomic datasets. As examples from the general Danish pig population, boar taint eQTLs [7] and fat mass [8] eQTLs have been identified. Typically, the sample sizes are limiting factors as transcriptomic profiling of various tissues is laborious and expensive compared to genotyping. There are several other challenges with eQTL analysis. If one wanted to map all possible SNP-gene pairs in a modern data set, which typically has thousands of expressed genes and at the minimum several tens of thousands of SNP, the total amount of tests will be at least in the order of 10^8^. This can pose computational challenges, but even worse, multiple testing problems. This is especially relevant as a cursory search of the Gene Expression Omnibus database (https://www.ncbi.nlm.nih.gov/geo/) for RNA-Seq studies reveals most studies having less than 100 samples. Therefore, it is important to have strategies for these issues when performing eQTL analysis. Example strategies used for filtering the expression data in previous studies include: filtering by estimated heritability of gene expression [9–11] or using only a limited set of genes[12].

Feed efficiency (FE) has been known for decades to be an important complex trait in pig breeding. Cost of feed is the largest economic burdens in commercial pig production [13, 14], and lower feed consumption leads to more environmentally friendly production. The two main metrics for feed efficiency are residual feed intake (RFI) [15] and feed conversion rate (FCR), which is the ratio between feed consumed and growth, with the latter being the most used in pig production. Selective breeding has improved FCR in pigs, but this has not led to direct gains in knowledge of the biological drivers of FE in pigs. Even with many studies being done on the subject, the genetic and biological background of FE in pigs is still not well understood [16]. The cost and difficulty of measuring FE likely plays into this, as it cannot be easily measured without expensive equipment and setup, unlike meat quality or litter size. FCR is the FE trait of choice in the Danish pig production. In this context, FCR is improved through a centralized breeding program where potential breeding sires are tested for efficiency via accurate measurements of feed intake and growth.

Muscle is the most important tissue in pig production in regards to economic value. Muscle plays a large role in energy metabolism and energy storage [17–19]. As such, there have been multiple studies on the muscle transcriptome in a FE context [13, 20–22]. While there are several eQTL studies performed in pig muscle [9, 12, 23, 24], there are none based on FE traits.

A connection between FE and mitochondria in muscle has been reported several times, in several species in the literature [13, 21, 22, 25–27]. The link between mitochondria and FE is well established, but the causal effect of mitochondria on FE is less clear. Jing and Vincent et al [13, 22] report lower mitochondrial expression in efficient pigs, while Gondret and Bottje et al [21, 26] report the opposite in pigs and broiler chicken. Given the evidence for mitochondrial effects, and the unclear causal nature of these effects, identifying genetic regulation of mitochondrial genes could assist in efforts to develop biomarkers for FE and further understanding of the functional effect of mitochondria on FE.

Trans-eQTLs are per definition distally located in relation to the genes they are affecting. This means that true trans eQTLs should have a mechanism that mediate correlations between expression and genetic variation. There has been evidence that trans-eQTLs can be mediated by local cis effects of the eQTL[28, 29]. One proposed method for the mediation is through cis affected transcription factors [29]. Given this evidence, we could hypothesize that the genes should be enriched for gene ontology categories that can interact with genomic context or regulate expression, as seen previously in genes near trans-eQTLs.

Here we performed cis and trans-eQTL analysis on a previously identified set of FCR related differentially expressed genes (DEG) and mitochondrial genes (known to be involved in energy metabolism and nutrient utilization), in a pig population comprised of Danbred Duroc and Landrace purebred pigs. The joint breed analysis provides genetic variations that can aid in the detection of eQTLs, as it has been proposed that natural genetic variation aids in eQTL analysis [30], and natural variation in FCR due to the breed differences increases the likelihood of identified eQTLs being relevant in an FE context. By focusing on DEG and mitochondrial genes, we applied a targeted approach for underpinning systems genetics of our phenotype of interest (FE). To improve the statistical and computational analysis, we reduced genotype input space through linkage disequilibrium (LD) and loci variation filtering. Finally, we tested the hypothesis that genes that were associated with SNPs identified as trans-eQTLs would belong to pathways that could mediate such effects, including expression regulation and DNA binding.

## Material and methods

### Sampling and sequencing

The pigs in this study were the intersection between the pigs genotyped in Banerjee et al. [31] and Carmelo et al [32], resulting in a selection of 38 pigs. All data processing steps follow those two studies, unless otherwise stated. Of the 38 male uncastrated pigs included in this study, 11 were purebred Danbred Duroc and 27 were purebred Danbred Landrace. The pigs were sent to the commercial breeding station at Bøgildgård, which is owned by the pig research Centre of the Danish Agriculture and Food Council (SEGES) at ~7kg of weight. The pigs were regularly weighed, and feed intake was measured based on a single feeder setup in a test period from ~28kg of weight until ~100kg. The period of measurement was determined by each pig’s commercial viability and a final weight of 100kg. All pigs were fed the exact same diet as reported in [33].

### Genotype data and filtering

DNA isolation from collected blood and genotyping was performed by GeneSeek (Neogen company - https://www.neogen.com/uk/). The Genotyping was based on the GGP Porcine HD array (GeneSeek, Scotland, UK), which includes 68,516 SNPs on 18 autosomes and both sex chromosomes. The SNPs were mapped to the *Sus scrofa* genome version 11.1 using the NCBI Genome Remapping from the *Sus scrofa* genome version 10.2. This was done using default settings. To insure that we had a sufficient representation of genotypes for each SNP, we used a MAF (minor allele frequency) threshold of 0.3. This removes SNPs that would be underpowered for the eQTL analysis and could not be related to expression changes due to lack of variation. It also had the advantage of reducing the overall testing space to a more conservatively sized set. This reduced the initial set of SNPs to a total of 27531. The next step performed was to remove groups of SNPs in high LD. To do this, we used the *LD_blocks* function from the *WISH-R* R package [34], which was applied with an R^2^ of 0.9. This grouped SNPs linearly across chromosomes into blocks based on a minimum pairwise R^2^ value of 0.9 between all SNPs in a block. After this step, 19179 SNPs remained. The genotypes were coded as 0 (homozygote major), 1 (heterozygote) and 2 (homozygote minor) for the eQTL analysis.

### Expression data, gene selection and filtering

Muscle tissue samples were extracted from the psoas major muscle immediately post slaughter, and the samples were kept at -25 C in RNA later (Ambion, Austin, Texas). The data was sequenced on the BGISEQ platform using the PE100 (pair end, 100bp length) with RNA extraction and sequencing performed by BGI Genomics (https://www.bgi.com/global/). The mean number of total reads was 64.5 million with standard deviation of 7.4 × ^10^. The mean number of uniquely mapped reads was 95.3% with a standard deviation of 0.33%. All reference genomes, gene annotation and analysis was based on *Sus scrofa* annotation version 11.1.96 from Ensembl. The reads were trimmed using Trimmomatic [35] version 0.39, with the default setting for paired end reads. Data QC was performed pre- and post-trimming using FastQC v0.11.9. Mapping was done with STAR aligner [36] version 2.7.1a adding the above mentioned *Sus Scrofa* ensemble annotation for splice site reference. Beside default parameters, the—quantMode GeneCounts setting was used for read quantification. The main interest was to investigate genes that could be related to FCR. We therefore based the set of genes on the methods in Carmelo et. al [32]. In brief, Differential Expression analysis (DEA) was performed using three different DE methods (Limma, EdgeR, Deseq2) [37–39] with FCR as the phenotype of interest. We then calculated the divergence between our observed p-value distribution for FCR and the uniform distribution for each method, enabling us to select a list of genes that are related to FCR. This was motivated by the fact that we had a large overrepresentation of low p-values in the DEA, meaning the distribution was anti-conservative. This resulted in a set of 853 genes. As mitochondrial genes have been implicated in FE in muscle in both our previous study and in several studies in multiple species [13, 21, 22, 25–27], we also selected all genes with a mitochondrial gene ontology (gene ontology id GO:0005739, N = 927) and included them in the analysis. The union of first 853 genes and the mitochondrial genes resulted in a set of 1772 genes. All genes were then filtered to have a minimum of 5 reads in at least 11 samples, as 11 was the size of the Duroc group. Testing revealed that genes with a single expression outlier could result in likely false positives. Therefore, all genes with a single expression value with a Z-score above 3 were removed, corresponding to a single observation with normalized expression further than 3 standard deviations from the mean. This resulted in a final gene set of 1425 genes.

### eQTL analysis

#### Calculation of eQTLs

All of the eQTL analysis was performed using R version 3.5.3. Gene expression was normalized using the *calcNormFactors* from the R package edgeR version 3.34.3 using weighted trimmed mean of M-values as the normalization method [40]. We performed eQTL analysis using the R package MatrixEQTL version 2.3[41], using R version 3.5.3. eQTLs were tested between all possible pairs between our genes (n = 1425) and genotypes (n = 19179). We added breed and batch effects as factors and RNA integrity values (RIN) and age (days) as covariates in the model. There were 5 levels of batch effect, representing different sampling days. Given that the samples were collected in slaughterhouse setting, it was necessary to include RIN in the model, but this should not be an issue if appropriately corrected for [42]. Breed and age have an effect on expression, as seen in our previous study [32] and thus must be accounted for. While the samples come from a selection of 28 different breeders in Denmark, there still was some relationship between pigs, especially if they came from the same breeder. Therefore, a kinship matrix based on 4 generations of pedigree was added as the error covariance matrix instead of using the default identity matrix. The cis-distance was set to 10^6^ bp. The analysis was done using both the *modelANOVA* (ANOVA) and the modelLINEAR (linear) options for both cis and trans-eQTLs, thus applying both a factor based model, and a linear model fit.

#### Statistical significance

To show the significance of the spike of low p-values we observed in the eQTL analysis (Fig 2), we performed bootstrapping by shuffling the genotype values of each SNP while maintaining the same expression values and covariates for each eQTL. This was done 1000 times, each time doing the full analysis for both the linear and ANOVA models with all possible genotype-gene pairs. We then calculated the number of random eQTLs with p-value < 0.01 for each sampling for both the cis- and trans-eQTLs. Assuming the shuffled values were normally distributed, we calculated the probability of observing our empirical number of p-values < 0.01. We also saved the lowest, the 10th lowest and the 100th lowest observed p-value for both trans and cis bootstrapped eQTLs for each iteration for comparison to the empirical values. Based on the empirical p-value distribution and bootstrapping analysis, pathway enrichment analysis was performed on the top putative eQTLs based on the results from the trans-eQTL linear model. The trans linear model was chosen over the trans ANOVA as the empirical p-value distribution for the ANOVA had an overweight of low and high p-values, which means that we should avoid using the overall distribution of p-values for conclusions, as there may have been issues with model assumptions. Thus, there was a risk of selecting models which violated model assumptions. In the linear version, the p-values were nearly uniform with a slight overweight of low p-values. This indicated a combination of non-significant eQTLs, which have uniformly distributed p-values under the null hypothesis, and a subset of true positives.

Mulitple testing correction was performed using FDR using the Benjamini-Hochberg method [43].

#### Orthonormalization

To visualize the expression and genotype values on the scale used by Matrix eQTL, we scaled and centered the design matrix of the covariates, the factors, the expression and the genotypes. Without this procedure, visualization of the relation between expression and genotype for a given eQTL would not be clear, as Matrix eQTL works and calculate p-values from this transformed scale. We used the *mlr*.*orthogonalize* function from the MatchLinReg package version 0.7.0 to orthogonilize the expression values and genotypes of each relevant gene and SNP in relation to the covariates and factors, using *normalize = True*. This procedure was done mimicking the method reported in the Matrix eQTL[41].

#### QTL regions and relation to FCR

To verify if our eQTLs were in known quantitative trait loci (QTL) regions, we first defined a region of 100kb upstream and downstream of each SNP as the SNP loci. The region size was conservatively defined based on reported haplotype block sizes in commercial pigs [44]. We then checked if the SNP coordinate had any overlaps with FCR quantitative trait loci (QTL) from the Pig QTL database[45]. We did the same procedure with the genes associated with each eQTL, except we did not extend the region beyond the gene boundaries.

#### Pathway analysis

We hypothesized that, if trans-eQTLs are not false positives, they should be enriched for functional categories that could relevantly cause distal interactions, in comparison to the background set of genes in the analysis. Therefore, to analyze the top trans-eQTLs, we calculated the number of additional empirically observed low p-values under 0.01, by subtracting the expected number of p-values < 0.01 given a uniform p-value distribution, from the observed number of p-values < 0.01. We then tested the enrichment of the genes in the top eQTL group for the following gene categories/ontologies: transcription factors(TF) (based on the AnimalTFDB 3.0 pig transcription factors [46]), DNA-binding (GO:0003677), DNA-binding transcription factor activity (GO:0003700) nucleus gene (GO:0005634), positive regulation of expression (GO:0010628), negative regulation of expression (GO:0010629) and membrane gene (GO:0016020). Each category was selected based on a biological hypothesis, with membrane gene serving as a control category. Membrane genes were selected as a control as we did not expect membrane genes to have enrichment of trans eQTL mediating effects. All GO terms were retrieved using biomart 2.42.0 with annotation from *Sus scrofa* 11.1. 96.

## Results

### eQTL analysis

**Fig 1:**
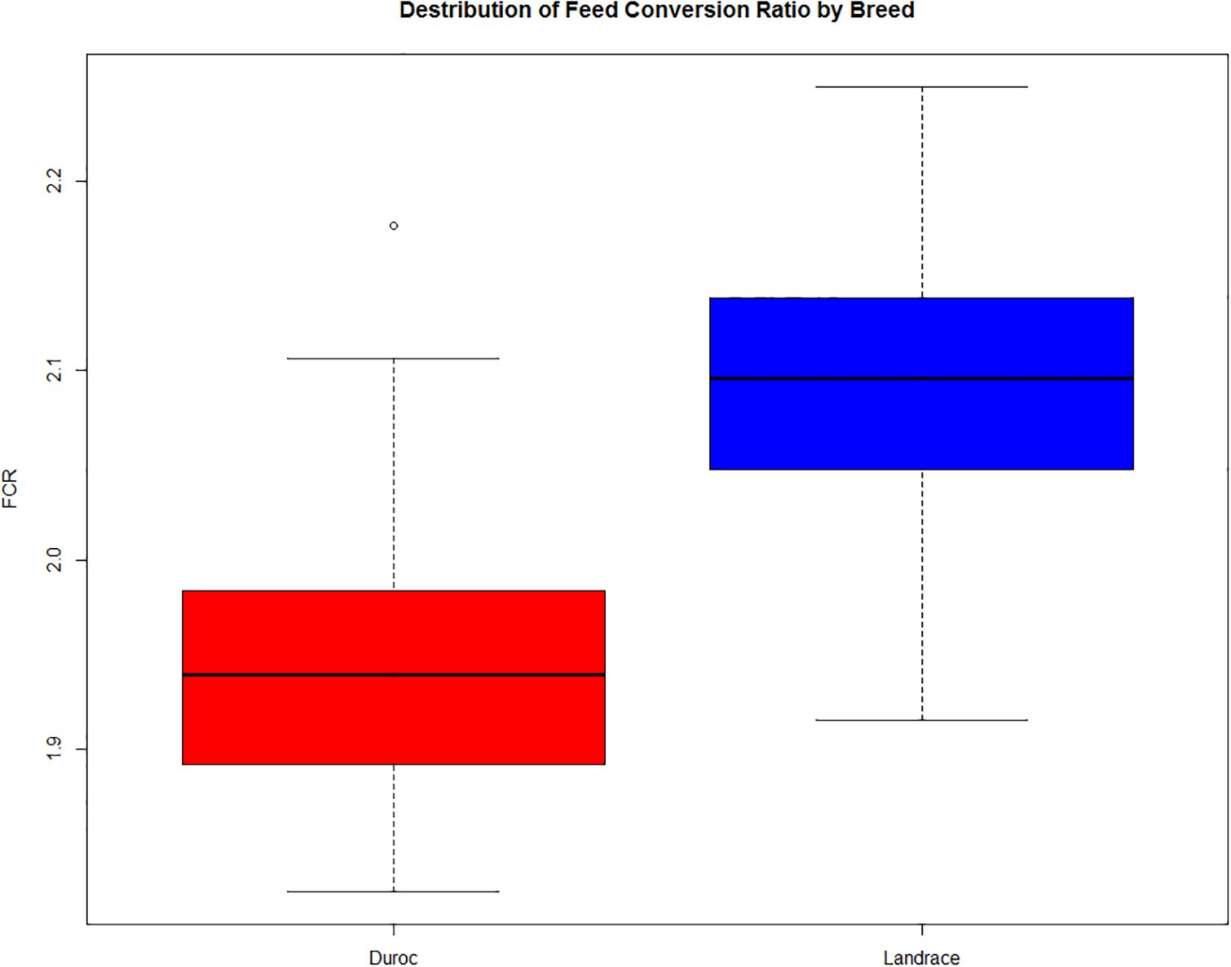
Distribution of FCR values in the two breeds. The Duroc pigs are generally more efficient, and thus have lower FCR on average.

**Fig 2:**
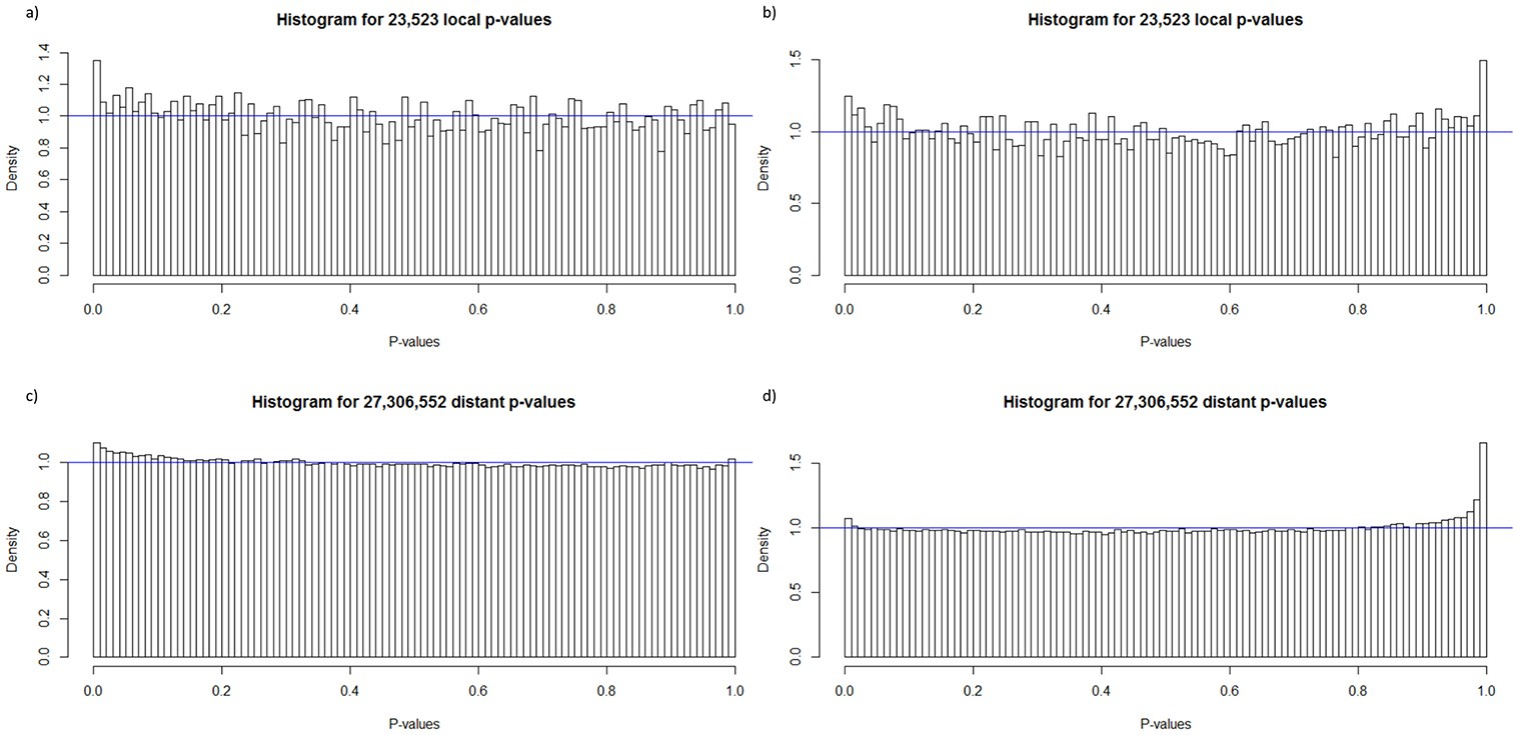
Histograms of the p-value distributions. Histograms of the p-value distributions of all cis (a,b) and trans(c,d) eQTL pairs in the linear(a,c) and ANOVA(b,d) models. Based on the overall distribution, we see a slight anti-conservative trend in the linear p-values in both cis and trans-eQTLs.

**Fig 3:**
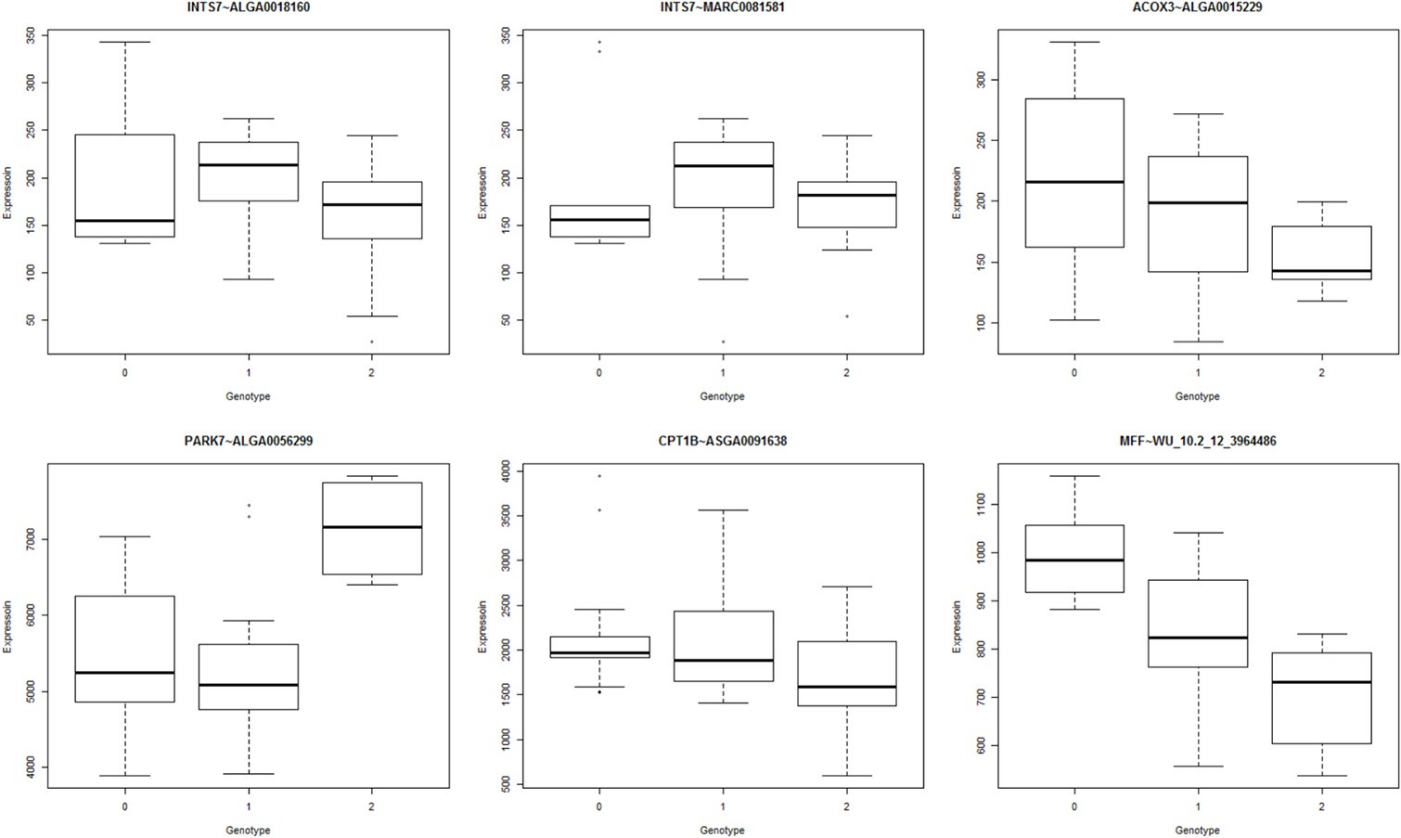
Top linear trans eQTLs. Boxplot of the top 6 trans-eQTLs from linear analysis. Comparing with the summary from Table 1, it seemed unexpected that the top left boxplot was the most significant eQTL. Overall, the 3^rd^ and the 6^th^ ranked eQTLs look visually more significant. This was because the genotypes and expression values were not corrected in relation to the model covariated and factors.

**Fig 4:**
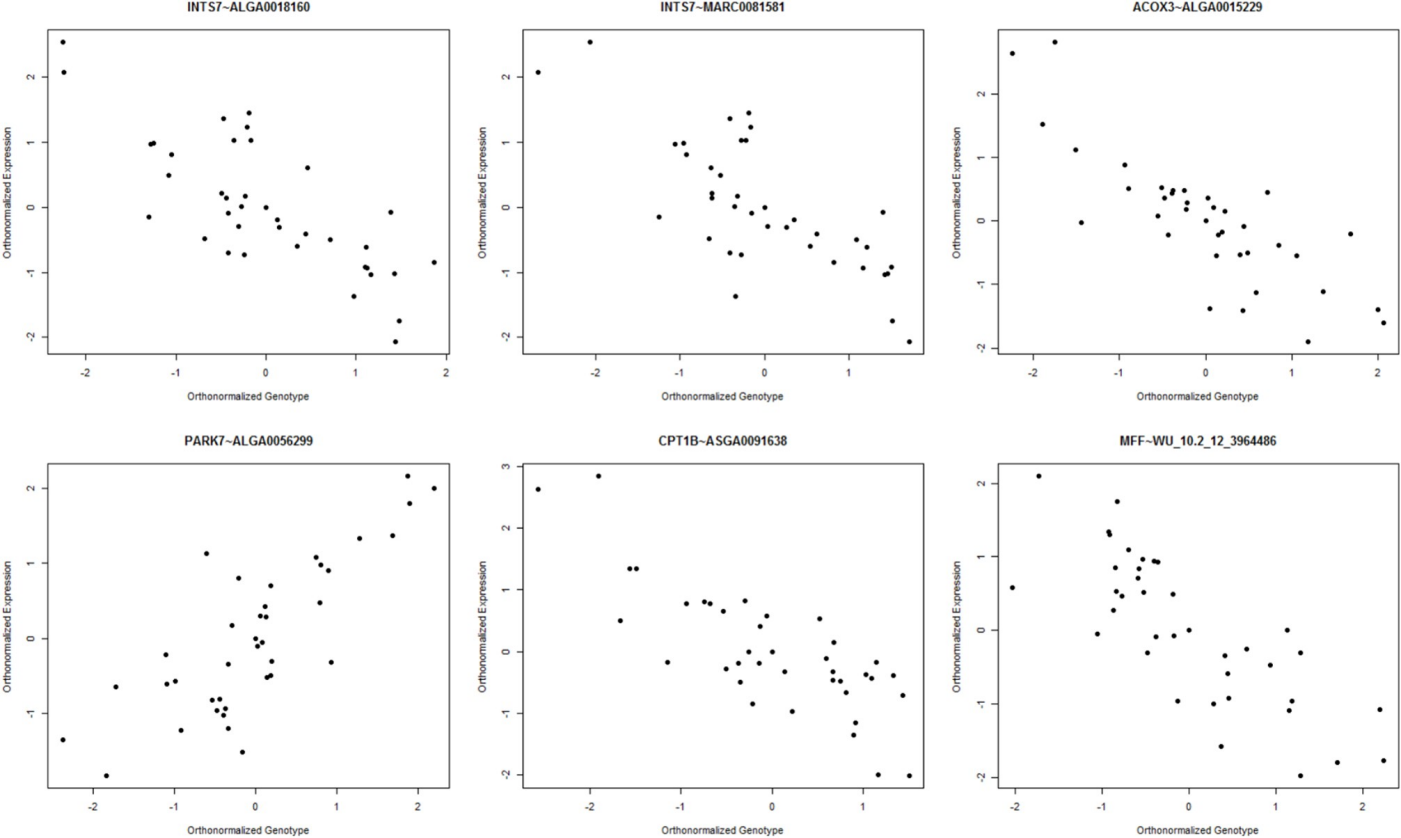
Orthonormalized expression and genotypes of top eQTLs. Scatter-plot of the orthonormalized expression and genotype values for the top 6 trans-eQTLs in the linear analysis. The linear relationship is quite clear on the transformed values, in comparison to the boxplots of the untransformed values.

To contextualize the eQTL analysis, it is important to understand the differences between the breeds. The Durocs are more efficient than the Landrace pigs, and thus have lower FCR (Fig 1). In eQTL analysis, the linear model was generally well behaved, with uniform p-values and a small increase of low p-values (Fig 2a and 2c). In the ANOVA model, we observed a spike of high p-values (Fig 2b and 2d), which may have been due to issues with model assumptions, but as we tested a large number of eQTLs, it was not practical to do model diagnostics on each eQTL. This did not mean individual ANOVA based eQTLs could not be valid, but we should be careful with drawing results based on the overall distribution. The cis-eQTLs had a more uneven overall distribution (Fig 2a and 2b), but this was likely due to the lower amount of tests combined with the histogram binning. At an FDR of 0.1, the only analysis that gave any significant results was the ANOVA analysis, which yielded 13 significant trans-eQTLs. In the trans linear analysis, due to the left skewing of the p-value distribution, all trans-eQTLs with p-value < 0.01 (N = 301213) had an FDR value of 0.9 or lower. This means that it was likely that we had true positive linear trans-eQTLs, we just lacked the power to identify them individually. Given this, and the results from the bootstrapping analysis (see below), we elected to present the top 10 eQTLs for each analysis, except for the ANOVA trans analysis, where we selected all with FDR < 0.1 (Table 1). This resulted in a p-value limit of 1.64 × 10^-7^ (FDR = 0.45) for the linear trans, a limit of 5.5 × 10^-5^ (FDR = 0.77) for the ANOVA cis, and a limit of 4.7 × 10^-5^ (FDR = 0.63) for the linear cis analysis. To further confirm the results, we visualized the top 6 eQTLs in the linear trans model (Fig 3), ordered from the lowest p-value (top left) to highest (bottom right). Given the low p-values reported (Table 1), the visualization, did not seem to support the results, which should show a linear relationship between expression and genotypes. The explanation is found in the way the Matrix eQTL implementation deals with the covariates and factors included in the analysis, or in our case, RIN, breed, batch and age. In Matrix eQTL, all covariates, expression and genotypes are centered and scaled, and the expression and genotype vectors are both orthogonized in relation to the covariate/factor matrix. Only after this step is the linear relationship between expression and genotype calculated. This is clearly seen once the same results are presented on the orthonormalized scale (Fig 4).

**Table 1:** Overview over the top cis and trans-eQTLs in all 4 four sub-analyses.

SNP name	Gene name	P-value	FDR	Chr	Position	Analysis
ASGA0047927	SLC20A2	7.25e-12	0.00020	10	44192068	ANOVA trans
H3GA0030144	SLC20A2	2.78e-11	0.00032	10	43919876	ANOVA trans
H3GA0030143	SLC20A2	4.40e-11	0.00032	10	43861540	ANOVA trans
ALGA0058821	SLC20A2	4.74e-11	0.00032	10	43913127	ANOVA trans
ALGA0043655	CSRNP1	2.07e-08	0.080	7	91867077	ANOVA trans
WU_10.2_15_121814208	CSRNP1	2.50e-08	0.080	15	110258817	ANOVA trans
M1GA0000775	CSRNP1	2.61e-08	0.080	1	13988391	ANOVA trans
ALGA0022682	KLF4	2.67e-08	0.080	4	7252911	ANOVA trans
ALGA0048662	ACLS1	2.99e-08	0.080	8	91823303	ANOVA trans
ALGA0067527	WDR81	3.520e-08	0.080	13	3403128	ANOVA trans
CASI0010058	ACLS1	3.54e-08	0.080	8	89930889	ANOVA trans
ASGA0089323	ACLS1	3.54e-08	0.080	8	89959199	ANOVA trans
ALGA0018160^1^	INTS7	4.88e-08	0.010	3	27307613	ANOVA trans
ALGA0018160^1^	INTS7	5.61e-09	0.15	3	27307613	Linear trans
MARC0081581^1^	INTS7	3.25e-08	0.31	3	27346598	Linear trans
ALGA0015229^2^	ACOX3	3.39e-08	0.31	2	116633408	Linear trans
ALGA0056299^2^	PARK7^1^	5.21e-08	0.36	10	1400269	Linear trans
ASGA0091638^2^	CPT1B	8.19e-08	0.38	4	626787	Linear trans
WU_10.2_12_3964486	MFF^1^	8.38e-08	0.38	12	4217210	Linear trans
ALGA0115669^2^^,^^3^	PARK7^1^	9.79e-08	0.38	10	1187360	Linear trans
DRGA0015709^1^	COMTD1	1.22e-07	0.42	16	2090820	Linear trans
ALGA0087901^1^	NSUN2	1.63e-07	0.45	15	129751572	Linear trans
WU_10.2_7_740616	Glycine N-phenylacetyltransferase	1.64e-07	0.45	7	618465	Linear trans
INRA0015708^1^	TBX3	0.00013	0.00013	14	37161034	ANOVA cis
WU_10.2_15_134661069	MY19	0.00015	0.77	12	38196853	ANOVA cis
WU_10.2_6_27531636	SYNPO2	0.00027	0.77	8	104924079	ANOVA cis
MARC0009689	IER5L	0.00030	0.77	1	268604956	ANOVA cis
WU_10.2_15_150992806	CRYM	0.00040	0.77	3	24920076	ANOVA cis
MARC0112128	CPT1A	0.00040	0.77	2	4531357	ANOVA cis
WU_10.2_15_91334711	ANKRD54	0.00050	0.77	5	10644697	ANOVA cis
WU_10.2_2_4374745	ANKRD54	0.00050	0.77	5	10687503	ANOVA cis
ALGA0019808^1^	RABPEK	0.00055	0.77	1	264970392	ANOVA cis
WU_10.2_3_18580686	SUCLA	0.00056	0.77	11	20630808	ANOVA cis
ASGA0054417	MYO19	0.00018	0.63	12	38196853	Linear cis
WU_10.2_X_128169493	RBMX	0.00018	0.63	X	112221790	Linear cis
WU_10.2_12_39624033	MYO19	0.00026	0.63	12	37981199	Linear cis
WU_10.2_3_183721	C7orf50	0.00031	0.63	3	335933	Linear cis
ALGA0108896^1^	CRYM	0.00035	0.63	3	24920076	Linear cis
ALGA0061099	MRPS31	0.00035	0.63	11	16202962	Linear cis
ALGA0061107	MRPS31	0.00035	0.63	11	16236530	Linear cis
ASGA0030240	NSUN4	0.00042	0.63	6	165835717	Linear cis
WU_10.2_14_153092095	ECHS1	0.00047	0.63	14	141129811	Linear cis
WU_10.2_14_153836231	ECHS1	0.00047	0.63	14	141357898	Linear cis

^1^Genes or SNPs in known FCR QTL regions.

^2^SNPs with p-value < 0.05 for linear association with FCR

^3^ SNPs found in top FCR modules from a previous study in the same pigs [47].

### Bootstrapping

**Fig 5:**
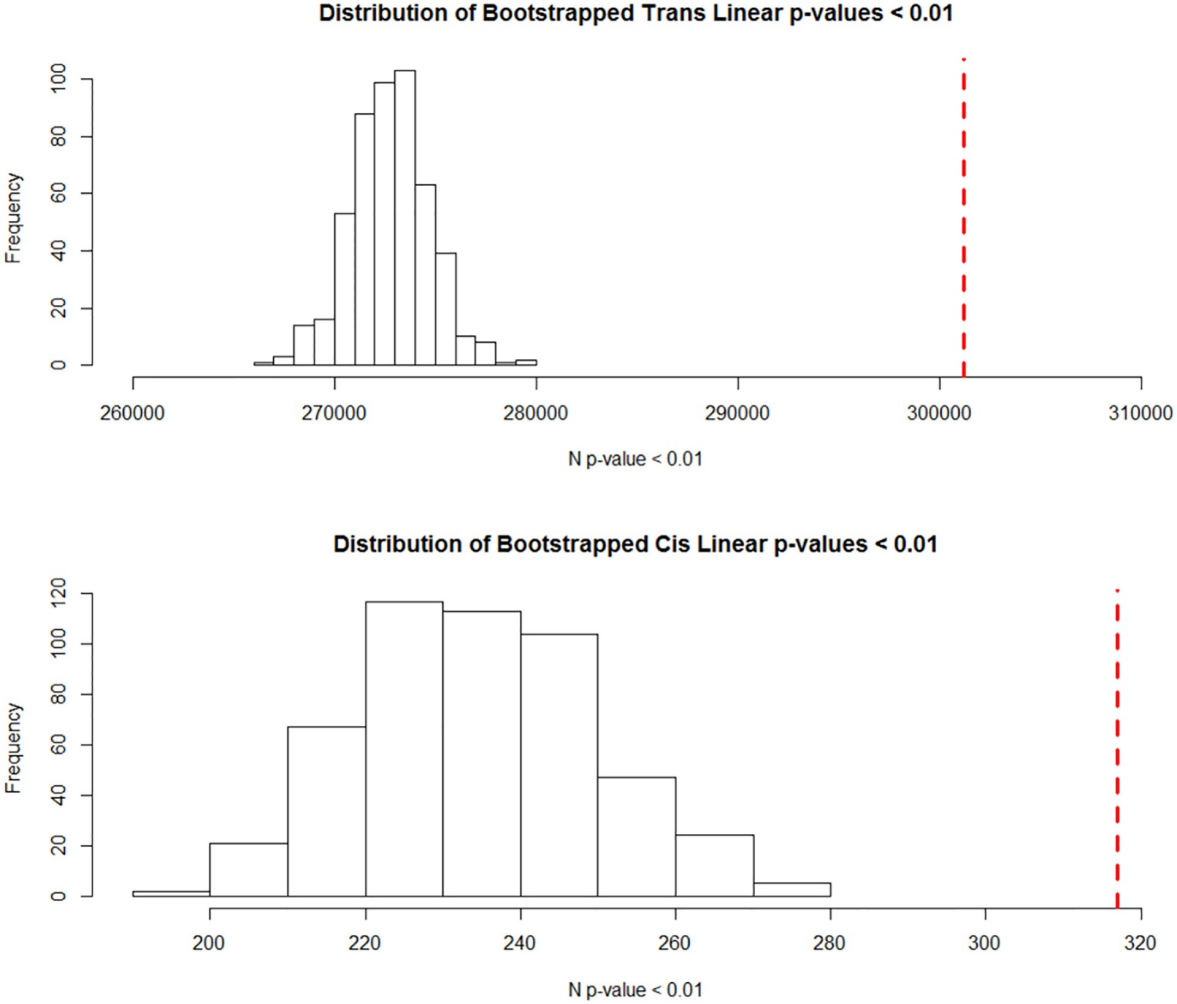
Distribution of bootstrapped low p-value counts. Histograms of the number of p-values below 0.01 in our 100 bootstrapped linear trans and cis-eQTLs analysis. The red dotted line represents the observed values. The likelihood of observing such extreme values by chance is essentially 0 in both cases, if we model the likelihood based of the normal distribution.

Bootstrapping is a useful tool when dealing with complex data, allowing us to get estimates of the likelihood of our observations without explicit probability calculations. Here, we wanted to show that our spike in low p-values in the linear analysis was statistically unlikely to happen by chance. Based on the bootstrapping, the probability of our observed number of p-values below 0.01 was essentially 0 if we model the distribution of p-values < 0.01 using the normal distribution (Fig 5). To understand why we did not have more significant results post-FDR, we compared the 1^st^, 10^th^ and 100^th^ p-values in our bootstrapped data with our empirical data (Table 2). The real data was more left skewed as we go down in in p-value rank. This indicated that the real data had lower bound on significance, but the overall results were not achievable by chance.

**Table 2:** Summary of comparison of empirical and bootstrapped data.

Model	Min P-value	10^th^ P-value	100^th^ P-value
ANOVA Cis	0.967	0.864	0.019
ANOVA Trans	0	0	0
Linear Cis	0.993	0.884	0
Linear Trans	0.184	0.028	0.014

Probability of observing a lower p-value than the lowest, 10^th^ lowest p-value and 100^th^ lowest p-values in our bootstrapping. In general, in relation to our random eQTLs, the empirical data was in the lower end of the bootstrapping, except in the linear cis analysis. It is interesting to note that by the 100^th^ p-value all the analysis outperform random data. This indicated that we did have true, but weak effects.

### Pathway enrichment analysis

As our genes were pre-selected, there was no *a-priori* reason to perform enrichment analysis. More exactly, there was no particular meaning in finding that the cis-eQTLs were enriched for some pathway. The cis-eQTLs are simply tests of correlation between local genomic context and expression, and significance denotes the identification of possible genetic expression regulatory mechanisms, so groups of cis-eQTLs do not have common underlying pathways. In contrast, for the trans-QTL, there are meaningful hypothesis one could state about pathways of genes affected by trans-eQTLs. Why would a gene have significant association to a distal genetic element? Previous studies have looked at the local context of trans-eQTLs [28, 29], however, we were not able to find any overrepresentation of our pathways in our local genes of top trans eQTLs data (S1 Table). Instead, we hypothesized that genes that interact with genomic context and/or regulate expression would be enriched in the low p-value group in comparison to the overall genes used in the trans eQTL analysis. We included genes that directly interact with genomic context, such as DNA binding genes, and regulatory genes, such as transcription factors and positive or negative expression regulators. To test our hypothesis, we selected the top 28147 SNP-gene pairs from our linear trans-eQTL analysis, which had a maximum overall FDR of 0.84. While it is clear that not all of these are significant, given the FDR values, there should be thousands of true positives in this set. In this top set of 28147 eQTLs, 1401 out of 1425 genes initially included in the analysis were involved in at least one eQTL, with the median number of eQTLs for each gene being 11 with a standard deviation of 53. This set represented our observed surplus of low p-values found when comparing with a uniform p-value distribution for eQTLs with a p-value < 0.01, motivated by our results from the bootstrapping (Fig 5). Traditionally, one might test our hypothesis using a pathway enrichment tool, but given that the eQTL data had a special structure, including repeated entries of the same genes from a smaller background set and a large overall number of input genes, it was not suiTable for typical methods. Instead, we used a more targeted approach, selecting specific categories we believed tested our hypothesis. The results from the enrichment, using the exact Fisher test, showed highly significant enrichment for DNA binding genes, transcription factors and DNA binding transcription factor activity (Table 3). All these categories fit our hypothesis, as they engage directly with distal genomic context. We also tested for nucleus genes, as we expected genes that are active in the nucleus to be more likely to interact with genomic context. Furthermore, we tested for general expression regulation, with the positive and negative expression regulation categories. Intriguingly, positive regulation was slightly depleted or unchanged, while negative expression regulation was the most enriched category. Finally, we included membrane genes as a control category that includes a large number of genes, as we did not believe they had a reason to be enriched, as membrane genes should primarily be active in the membrane, and not mediating expression in the nucleus. In comparison to the set of genes we used in the eQTL analysis, we did not find enrichment in the membrane genes. As a control of the enrichment, we also compared with all expressed genes in our samples, beyond our selected eQTL analysis set. This aids in the interpretation, and acts as a safeguard, as if there was high divergence in the two comparisons the results might just be an artefact of our methodology. We see similar results comparing with all expressed genes, and due to the large number of genes in both the expressed set and the trans-eQTLs, we get very significant p-values. When comparing to the expressed gene-set, we do see slight enrichment for membrane genes. It should be noted that the baseline ratio between our test set and expressed set for membrane genes is already slightly enriched, at 1.05 (see S2 Table), and small effect sizes are significant with the large number of genes included from the trans eQTL genes and the expressed set of genes.

**Table 3:** Pathway enrichment results.

Category	N Genes	Fold Enrichment compared to Background	P-value	Fold Enrichment compared to expressed genes	P-value
**Transcription Factor (Pig TF database)**	3145	2.27	1.0x10^-13^	1.40	<2.2x10^-16^
**DNA binding (GO:0003677)**	3394	2.20	8.9x10^-14^	1.73	<2.2x10^-16^
**DNA-binding transcription factor activity (GO:0003700)**	2721	3.36	<2.2x10^-16^	2.36	<2.2x10^-16^
**Positive regulation of expression (GO:0010628)**	346	0.67	0.07	0.67	8.9x10^-6^
**Negative regulation of expression (GO:0010629)**	1887	4.34	<2.2x10^-16^	5.39	<2.2x10^-16^
**Nucleus gene (GO:0005634)**	8811	1.33	2.4x10^-6^	1.18	1.8x10^-12^
**Membrane gene (GO:0016020)**	7707	1.06	0.30	1.15	1.8x10^-8^

Enrichment analysis based on the linear trans eQTLs with p-value < 0.01, based on the Fisher exact test. N genes denotes the number of eQTLs which matched each category from the top linear trans eQTL set (total N = 28147). The enrichment calculated comparing with the original input set of 1425 genes (column 4 and 5), and to the set of expressed genes (N = 13202) in our muscle samples for additional comparison column (5 and 6).

## Discussion

In this study, we applied Matrix eQTL to a set of genes previously identified as having potential relations to FCR. We presented the top results of both cis and trans-eQTLs based on a linear association and a factor based analysis (ANOVA). There have been several muscle eQTL studies in pig before [9, 12, 24, 48–52]. However, direct comparison of our results to previous studies is not straight forward, for several reasons. None of the other studies were applied to FCR, and as the genes and SNPs selected in each study were generally selected based on the phenotype of interest, this limits the overlap. Furthermore, due to the statistical challenges, many divergent strategies were employed, for example using a pre-GWAS[49], picking a limited set of pathway specific genes [12] or using a limited set of microsatellites[52]. Some studies included heritability analysis as a filter [9],but this may be a flawed strategy as it has been shown that this might filter out potential eQTLs, especially as trans-eQTLs show low overall heritability [24]. The studies above include both crossed, purebred and F2 half-sib pig populations, in contrast to the two-breed model applied here. Given all these factors, and the novelty of FCR in an eQTL context, we could not compare our study very specifically to others, and one should thus view our study as a pilot study for FCR eQTLs. When comparing with our own previous study of genetic networks in the same population [47], we found that two of our top eQTL snps from the trans linear model and ANOVA trans model, ALGA0115669, was also found in a SNP module highly correlated to FCR. Interestingly, ALGA0115669 has a p-value < 0.05 for linear relationship to FCR, and its associated linear trans eQTL gene, PARK7, is located in a known FCR QTL region.

We included two pure breeds in the analysis, Duroc and Landrace, which is an unusual choice when reviewing the literature presented above. Many studies published have inbred lines, but it has been suggested that it would be advantageous to do eQTL analysis on a natural genetically varying population [30], such as two separate breeds. For the input SNPs, we made several choices for maximizing the number of relevant SNPs to include. First, we selected a quite high cutoff of 0.3 MAF. This allowed us to have high enough variation at each included SNP to fit the eQTL models, given our low sample size. It has also been shown, that in chip-based data such as ours, the overall structure in the data is robust to different MAF cutoffs [53], thus this should not impart any biases into the results. Finally, we grouped SNPs in high LD (R^2^ >0.9) into blocks and used tagging variants to represent blocks. This allowed us to reduce the space further, removing redundant genetic information, thus relaxing our multiple testing thresholds. We chose a concervative cis-eQTL distance of 1Mb, which is on the lower end for pig studies [24]. Given our relatively low samples size we wanted to keep the cis analysis as conservative as possible.

In the individual eQTLs results, one should be careful with over interpreting, but instead view the eQTLs as candidates for further study. Based on a qualitative analysis, we did find several interesting genes among the top eQTL candidates. We identified two genes associated to meat quality in previous pigs studies, namely SLC20A2 (Solute Carrier Family 20 Member 1) in Durocs [54] and INTS7 (Integrator Complex Subunit 7) in Chinese pigs [55].

Two genes were identified to be related to response to feed intake: CSRNP1 (Cysteine And Serine Rich Nuclear Protein 1) was found to be a metabolic response gene in relation to feed intake in Durocs [56]; and CPT1A (carnitine O-palmitoyltransferase I) was differentially expressed depending on diet in pigs [57]. CPT1A was also related to reproduction traits [58], with the IER5L (Immediate Early Response 5 Like) and Tbx3 (T-box 3) being related to teat number and mammary glands development, respectively [59] [60]. These last three genes are interesting as only the less efficient pigs, the Landarace pigs, were selected for traits related to reproduction and caring of piglets.

Several genes were related to fat and fat metabolism. The ACOX3 (acyl-CoA oxidase 2) gene, a fatty acid metabolism gene, had previous cis-eQTLs identified associated with it [12]. CPT1B (arnitine palmitoyl transferase 1B), was differentially expressed in large whites versus an indigenous high-fat breed [61]. This last one is interesting as both CPT1B and CPT1A show up in our analysis. ACSL1 (Acyl coenzyme A long-chain 1 synthetase), which is a key gene for animal fat synthesis and fatty acid beta-oxidation, has been found to be differentially expressed in multiple tissues, including muscle, between different pigs breeds[62]. The same study also identified genetic variants with breed specific allele frequencies in the ACLS1 flanking region, concluding that ACLS1 might be important for breed specific fat deposition and meat quality. Finally the KLF4 gene (Krüppel-like factor 4) is an essential regulator of adipogenesis [63].

Beyond the small gene categories from above, we also found several other interesting genes. The PARK7 gene, a gene that codes for a protein that protects from oxidative stress[64], does not appear in a pig related context in the literature, but it is found in a known FCR QTL region, and its eQTL SNP was found in a module highly correlated to FE in our previous work [47]. MFF was similarly found in a known FCR QTL region. Myosin XIX (MYO19) was a candidate gene for eating behavior traits due to a nearby significant SNP in the same Duroc population our pigs come from [65]. The Uncharacterized Protein C7orf50 had a previous cis-eQTLs identified in a behavioral context in humans[66]. The SYNPO2 (Synaptopodin 2) gene has previously been found to be differentially expressed between Yorkshire and Wannanhua pigs in muscle [67]. The SUCLA2 (Succinate-CoA Ligase ADP-Forming Subunit Beta) gene has polymorphisms which have been associated with growth in pigs [68].

While the above-mentioned genes might seem like a mixed group of results, the main takeaway was that each of the genes mentioned above had appeared in previous contexts that demonstrated breed variation, genetic regulation and association with traits under selection in production pigs, thus giving qualitative evidence that increases the likelihood of the eQTLs being true positives.

The final and perhaps most interesting result in our analysis stemmed from the enrichment analysis in the linear trans-eQTL analysis. We initially hypothesized that we would find enrichment for genes that interact with genomic context and expression regulating genes. The findings, and their significance level, showed a strong overrepresentation of DNA-binding genes, DNA-binding with transcription factor activity genes and transcription factors. These results have a quite straightforward interpretation—genes that interact on a genomic level had a higher chance of having trans-eQTL activity. This could be mediated through direct interactions, or through indirect effects, such as transcription factors acting on each other, thus mediating their own genetic effect to other genes. The more intriguing result is the contrast between negative and positive gene regulation. It is possible that a gene regulation mechanism correlated to trans-eQTLs explains why we have such a high enrichment of negative regulation, but given our sample size and study power, it is difficult to assess individual genes, and thus properly grasp specific interpretation of these results. One possible cause of the negative gene regulation enrichment could be the activation of apoptotic pathways in the cells post mortem, as apoptotic processes are observed in muscle cells post-mortem [69]. The pathway results were highly statistically significant, indicating that these effects should be observable in other eQTL studies. In general, given the complexity of gene expression regulation, further study is needed before we have a proper understanding of the contrast between negative and positive expression, and the rest of the enrichment results. Based on our analysis, we propose that these enrichments could be general patterns of trans eQTLs due to the implications of long distance expression mediation effects. Pathway information of putatitive eQTLs could guide us in the validation of true trans-eQTLs. Essentially, identification of such biologically relevant effects can be used as an extra layer of evidence for true positive trans-eQTLs. If we view these results in an animal breeding and selection context, it shows that there may be a merit to weight the importance of genetic variation based on the gene and pathway context, and weighted methods have been shown to improve the accuracy of breeding value estimates [70, 71].

## Conclusion

FE is a challenging phenotype to study, as it is complex and affected by many factors, such as metabolism, growth and activity level. Furthermore, testing for FE is expensive, as it requires costly equipment to measure feed intake of individual animals, making FE biomarkers valuable. The analysis of eQTLs is a statistically challenging but powerful method for the functional analysis of genetic variation. Our final eQTL analyses involved a total of 1 2.7x10^7^ Gene-SNP combinations. The 1425 genes were selected from the results of RNA-Seq based differential gene expression analyses using 25880 genes related to FE and genes related to mitochondrial activity and the 19179 SNPs were from GGP Porcine HD 70k SNP array. We used 1000-fold bootstrapping and gene set enrichment analysis to further validate and analyze our detected eQTLs. By using pigs from two breeds with different selection goals, and these analytical approaches, we were able to identify putative cis and trans-eQTLs (N = 13, FDR<0.1). Qualitative analysis revealed several eQTLs genes associated to traits and pathways highly relevant for pig breeding and variation in pigs. We identified highly significant enrichment of regulatory and DNA binding genes in trans-eQTLs. This result provided a strong evidence for the validity of our trans-eQTLs, and evidence for a general hypothesis of the nature of trans-eQTLs.

## Supporting information

S1 Table Pathway enrichment of eQTL gene set. Fisher exact test for selected pathway enrichment of our genes included in the eQTL analysis and our set of total expressed genes.
(DOCX)

S2 Table Pathway enrichment of trans eQTL local genes. Fisher exact test for selected pathway enrichment for the nearest genes to our trans-eQTLs with P < 0.01 compared with our set of total expressed genes.
(DOCX)
