# Supplementary material for: Genetic variations (eQTLs) in muscle transcriptome and mitochondrial genes, and trans-eQTL molecular pathways in feed efficiency from Danish breeding pigs
Source: PLoS One. 2020 Sep 17;15(9):e0239143. doi: 10.1371/journal.pone.0239143 (PMC7498092; doi:10.1371/journal.pone.0239143)
Supplement: S1 Table — Fisher exact test for selected pathway enrichment of our genes included in the eQTL analysis and our set of total expressed genes. (DOCX) [file pone.0239143.s001.docx]

| **Category** | **Fold Change** | **P-value** |
| --- | --- | --- |
| *Transcription Factor* | 0.83 | 6.1x10^-6^ |
| *DNA binding* | 0.86 | 5.3x10^-4^ |
| *DNA-binding transcription factor activity* | 1.05 | 0.37 |
| *Positive regulation of expression* | 1.11 | 0.20 |
| *Negative regulation of expression* | 1.20 | 0.056 |
| *Nucleus gene* | 0.70 | <2.2x10^-16^ |
| *Membrane gene* | 1.02 | 0.31 |

***Table S2 Pathway enrichment of trans eQTL local genes*** *– Fisher exact test for selected pathway enrichment for the nearest genes to our trans-eQTLs with P < 0.01 compared with our set of total expressed genes.*
