# Supplementary material for: Genetic variations (eQTLs) in muscle transcriptome and mitochondrial genes, and trans-eQTL molecular pathways in feed efficiency from Danish breeding pigs
Source: PLoS One. 2020 Sep 17;15(9):e0239143. doi: 10.1371/journal.pone.0239143 (PMC7498092; doi:10.1371/journal.pone.0239143)
Supplement: S2 Table — Fisher exact test for selected pathway enrichment for the nearest genes to our trans-eQTLs with P < 0.01 compared with our set of total expressed genes. (DOCX) [file pone.0239143.s002.docx]

| **Category** | **N genes in category** | **Odds Ratio** | **P-value** |
| --- | --- | --- | --- |
| *Transcription Factor* | 70 | 0.62 | 8.4x10^-5^ |
| *DNA binding* | 77 | 0.83 | 0.14 |
| *DNA-binding transcription factor activity* | 38 | 0.72 | 0.042 |
| *Positive regulation of expression* | 25 | 0.98 | 1 |
| *Negative regulation of expression* | 22 | 1.33 | 0.20 |
| *Nucleus gene* | 310 | 0.97 | 0.70 |
| *Membrane gene* | 360 | 1.04 | 0.55 |

***Table S2 Pathway enrichment of eQTL gene set*** *– Fisher exact test for selected pathway enrichment of our genes included in the eQTL analysis and our set of total expressed genes.*
